# Adverse Events due to Lack of Precision in Total Hip Arthroplasty: The Potential of Provocation-Based CT for Diagnosis of Implant Loosening

**DOI:** 10.1155/2021/8836687

**Published:** 2021-06-02

**Authors:** Olof Sandberg, Henrik Olivecrona, Pelle Gustafson

**Affiliations:** ^1^Sectra, Linköping, Sweden; ^2^Karolinska Institute, Solna, Sweden; ^3^Landstingens Ömsesidiga Försäkringsbolag, Stockholm, Sweden

## Abstract

Adverse events in total hip replacement (THR) may have several origins, one being lack of precision in diagnosis and/or during surgery. This study describes the pattern and frequency of avoidable injuries in THR and the potential value of a new tool for early diagnosis of implant loosening. This retrospective study was based on all (*n* = 1 456) settled claims regarding THR in the Swedish National Patient Insurance database from 2010 to 2017. The claims and medical records were analyzed for root causes, with special focus on adverse events where lack of precision could be the cause. In a second stage, we assessed in 10 patients (20 implants) the diagnostic precision of a new software tool based on provocation-CT. These were all patients where the implant loosening diagnosis was deemed as inconclusive after a first plain X-ray. The findings from the provocation-CT and plain X-ray were compared to the surgical findings at revision. While 3 of 20 implants were correctly diagnosed with plain X-ray, for dynamic CT, this number was 14 of 20 implants. The retrospective study showed that the most common types of injuries were infections (34%), nerve injury (29%), mechanical problems (14.5%), dislocation (6%), and miscellaneous complications (16.5%). Of the patients with mechanical complications, one-third had aseptic implant loosening. Statement of clinical significance: we estimated that almost 200 patients in our registry study had mechanical complications after THR, mainly implant loosening. A third of these could potentially have been diagnosed and treated if CT examination with a dedicated image analysis tool to assess implant loosening after THR had been available.

## 1. Introduction

In an absolute majority of total hip replacements (THRs), precision is already today enough to give a good outcome. However, considering the number of THRs, even a low percentage of adverse events results in a large number of individuals affected by these adverse events.

A recent survey of adverse events in Swedish orthopedic care, based on 950 adverse events in 733 patients and 4 994 admissions, showed that surgical and other invasive adverse events constituted 14% of all adverse events [1]. Aseptic prosthetic loosening is the most common cause for revision of THR, according to the yearly reports from the Swedish Hip Arthroplasty Registry [2].

Surgical adverse events are the type of adverse event that, at least theoretically and in comparison, with other types of adverse events, should be relatively easy to describe, address, measure, and solve. However, a deeper understanding of adverse events than just describing their frequency is needed to solve the problem. Prosthetic loosening is an area where increased precision has potential benefits. We have previously demonstrated the possibilities of a more precise assessment of implant position and loosening in THR using computer tomography (CT) examinations [3–6].

As part of a large Swedish research initiative to improve precision in orthopedic surgery, PRECIIS, we set out to identify causes for avoidable injuries after THR surgery, especially those where lack of precision, either during surgery or in diagnosing, was deemed as the causing factor. We also wanted to see if a diagnostic tool with increased precision, provocation-CT (IMA), could have helped to better detect implant loosening after THR.

This is the first study of the pattern of avoidable injuries due to lack of precision after THR using the LÖF database. The intention was to describe the magnitude of the problem and identify potential solutions.

## 2. Methods

The Swedish Patient Injury Act makes it possible for a patient who has experienced a suspected or obvious injury or complication during care to file a claim with the Swedish National Patient Insurance Company (LÖF) for investigation and possible redress. If the adverse event is deemed avoidable according to the insurance terms, the patient is compensated for actual and expected financial losses. The insurance company holds a database over all filed claims since 1995, making it possible to analyze patterns and causes of adverse events.

### 2.1. Registry Study

**Figure 1:**
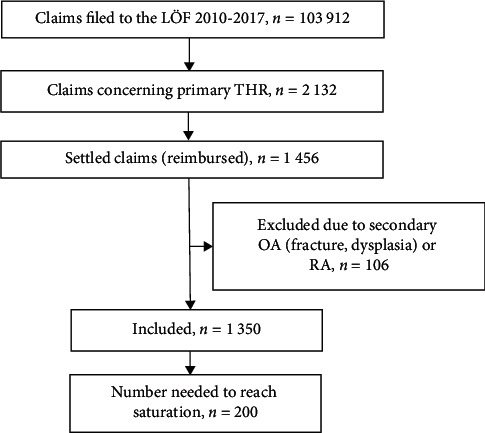
Selection of the study population.

From 1 January 2010 to 30 June 2017, 103 912 claims were filed with the LÖF, of which 2 132 concerned all types of adverse events after primary THR. These claims were selected for this series. Of the claims that were settled and led to reimbursement (were considered as avoidable), 1,456 claims concerned a primary hip replacement. 106 patients with secondary hip OA due to fracture and dysplasia and patients with rheumatoid arthritis were excluded, leaving 1,350 patients for analysis. 1,348 THRs were performed due to primary hip OA (osteoarthritis) and 2 due to ON (Aseptic Idiopathic Osteonecrosis, *n* = 2) (Figure 1).

As we were only interested in the type, pattern, and avoidability of adverse events and the database does not permit estimation of specific incidence figures, we initially reviewed every 10^th^ medical record of the 1 350 that fulfilled the inclusion criteria for types and pattern of adverse events until we had reached a subset of 100 patients and reviewed their individual records in detail. In order to avoid bias due to different treatments during different periods of time, we made the list of patients randomized as to date instead of sorting by age (which is not linked to date), a subset of 100 patients based on every 10^th^ medical record. As the types, pattern, and frequency did not differ between the 2 subsets and no new types or patterns emerged in the second subset, we considered the data as saturated and undertook no further inclusion. In all, we, thus, reviewed 200 of the original 1 350 claims until we had a sample of 200 patients with avoidable injuries. This cohort of 200 patients was then analyzed for basic descriptive data and for adverse events related to the THR surgery.

We recorded the following adverse events:

  Infection (defined according to [7]) defined as (1) there is a sinus tract communicating with the prosthesis or (2) a pathogen is isolated by culture from at least two separate tissue or fluid samples obtained from the affected prosthetic joint or (3) four of the following six criteria exist: (a) elevated serum erythrocyte sedimentation rate (ESR) and serum C-reactive protein (CRP) concentration, (b) elevated synovial leukocyte count, (c) elevated synovial neutrophil percentage (PMN %), (d) the presence of purulence in the affected joint, and (e) isolation of a microorganism in one culture of periprosthetic tissue or fluid.
  Recurrent dislocation of the prosthesis defined as the hip prosthesis dislocating more than 3 times and having occurred more than 3 months after surgery.
  Nerve injuries including injuries to the sciatic nerve, as well as Trendelenburg limp due to m. gluteus insufficiency and due to injury to n. gluteus superior.
  Mechanical complications defined as aseptic prosthetic loosening of one or more of the components, excessive wear of one or more of the components, intraoperative fractures, implantation of either missized components or incompatible components, misplaced components, leg-length discrepancies, and implant fractures.
  Miscellaneous complications defined as other adverse events related to the THR procedure causing a reason for reimbursement such as operation with resurfacing hip replacement that had been withdrawn from the market, urinary tract infection with bladder dysfunction due to catheter after surgery, and decubitus pressure ulcers. These complications were not further studied, as they were not considered as due to lack of precision.

### 2.2. Provocation-CT Method for Evaluating of Implant Loosening

**Figure 2:**
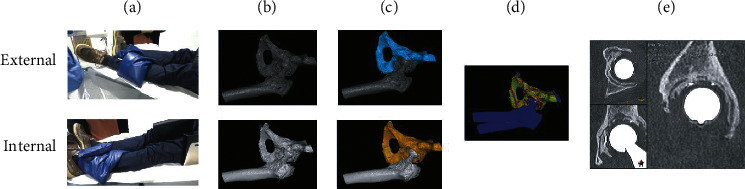
Workflow for (a) image acquisition, (b–d) processing, and (e) visualization. The moving image overlay is a key feature and can be found under the supplementary material. (a) Patient placement. (b) CT volume. (c) Subvolume. (d) Alignment. (e) Video visualization.

The provocation-CT method used for evaluation of implant loosening comprised evaluation of paired CT volumes acquired in the supine position, one volume with the affected leg in maximum external rotation and one with the leg in maximal internal rotation. This created a torsional stress over the implant. The CT volumes were evaluated for loosening using one of two tools for volume registration. The first patients were analyzed with software developed at our department and the latter part of patients with a commercial grade tool built on the experiences from the older one. Both tools allow the user to register either a prosthetic component or the surrounding bone and view the registered volumes in overlay images. Loosening of the implant is detected as a shift in the position of a prosthetic component relative to the surrounding bone between the external and internal rotation CT volume. Further details of this method have also been previously described elsewhere [3, 5, 8]. A patient has his/her leg externally rotated and locked in place with a sandbag and CT of the hip taken. Thereafter, the leg is rotated internally and locked in place with a sandbag and another CT of the hip taken (Figure 2(a)). These images are then loaded into image registration software where a subvolume is selected (Figure 2(c)) and aligned (Figure 2(d)). This produces an overlay moving image locking onto the pelvis and showing if cup movement relative to the pelvis was induced by the internal/external rotation (see the supplementary video). The matching is repeated for the femur to visualize any stem movement.

Sixty patients from our department and other orthopedic departments in Stockholm who were referred to us for provocation-CT during 2016 for suspected implant loosening have been examined. This cohort is separate from the registry study cohort. They were selected because of difficulties to establish a conclusive diagnosis based on standard radiographic assessments. Ten of these patients that had been examined with plain X-ray examination and provocation-CT-based examination and had undergone surgery gave their informed consent for us to review their records and radiographs for this study. In 9 patients, implant loosening of the cup or stem after THR was suspected and in one implant loosening of the femoral component after operation with a tumor knee prosthesis (METZ) was suspected. The findings on standard plain X-ray examination and provocation-CT were compared to the surgical findings that served as the true result. The results of the standard plain X-ray examination and the provocation-CT for the implants were grouped in the following categories: “correct result” if implant loosening found in the examination also was found in surgery, “inconclusive findings” if implant loosening could not be determined in the examination prior to surgery, or “incorrect result” if the implant was diagnosed as loose but found to be stable in surgery or vice versa.

### 2.3. Ethics

For the registry study, the Stockholm Ethics Committee granted permission (2017/1737-31) For the IMA part of the study, the same committee judged this as quality control (2017/1792–31/2), and hence, no permission was needed.

## 3. Results

### 3.1. Registry Study

One hundred and thirteen of the 200 patients were women. The mean age was 66 (39–93) years. Sixty-eight patients received reimbursement due to infection, 12 due to recurrent dislocations, 58 due to nerve injuries including Trendelenburg limp due to m. gluteus insufficiency, 29 patients due to mechanical complications, and 33 patients due to miscellaneous complications (Table 1).

Of the 29 patients with mechanical complications, there were 12 patients with aseptic loosening, three in combination with prosthetic stem fracture. In seven of these patients, the clinical signs of prosthetic loosening had started more than one year before the diagnosis was established. There was one case of excessive wear, in four cases, the wrong prosthetic size was used, in four cases, the implant was malpositioned, in two cases, there was leg length discrepancy, and in six cases, there were intraoperative fractures. The most common complication was implant loosening in 12 patients.

### 3.2. Review of Patients Examined with Provocation-CT for Evaluating of Implant Loosening

Ten patients had been examined with standard plain X-ray examination and provocation-CT examination and also undergone surgery. This group consisted of 4 women and 6 men, mean age 57 years, who had previously been operated for hip osteoarthritis (*n* = 7), rheumatoid arthritis (*n* = 1), and a primary bone tumor (*n* = 2). The primary surgery had been a cemented THR in seven cases and an uncemented THR in two cases and a cemented tumor knee replacement in one case. The standard X-ray approach led to a correct result according to surgical findings during revision in 3 of the 20 implants, compared to 14 of the 20 implants examined with provocation-CT (Tables 2 and 3).

## 4. Discussion

In Sweden, around 17,000 total hip replacements (THRs) are performed annually, and 97% of all THRs performed during the last 10 years were not followed by a reoperation [2]. However remarkable this achievement is, it still leaves some 500 Swedish patients with a need for reoperation after primary THR.

The incidence of postoperative complications is still the most frequently used surrogate marker of quality in surgery. A complication can be defined as “any deviation from the ideal postoperative course that is not inherent in the procedure and does not comprise failure to cure” [9].

The findings of our registry study indicate that a third of THR-related mechanical complications could potentially have been diagnosed better with a more precise tool. If our sample of 200 patients in the study cohort of 1,350 was representative, almost 200 of the 1,350 patients can be estimated to have suffered from mechanical complications that would have been reimbursed and in approximately 55 patients IMA would have contributed to an earlier and more correct diagnosis, potentially avoiding a lengthy and resource demanding diagnostic process.

A strength of this study was that it only includes patients with injuries that have been confirmed as avoidable. As the LöF has access to the complete medical records of the patients, we could do an in depth-assessment of each patient. The definition of avoidable injury was according to the insurance terms, which are based on the Patient Injury Act (ref SFS 1996 : 799). According to this act, “compensation is allowed for an injury caused by examination, care, treatment, or similar procedure, provided that the injury could have been avoided by choice of a different manner of performance or by a different procedure with the same effect, but with a lower total risk.” Evaluation is performed in retrospect. The experienced professional is considered as the norm, which, for the topic of this paper, can be equalled with that if the THR, or the diagnostic work-up, was performed in accordance with what an experienced surgeon following professional standards at the time of surgery would have done, the injury is most likely not compensated. A limitation of this study is that as the LöF only comprises claims that have been submitted voluntarily by patients, this study cannot make any statements about incidence or prevalence in general. A further limitation is that the decision on the injury being avoidable or not is taken by someone not involved in the care of the patient and, hence, has no access to all details of the care. However, the insurance terms are clear, and the evaluation is performed by experienced orthopedic surgeons not involved in the care of the patient, factors that all speak in favor of an objective evaluation.

We also reviewed a cohort of selected cases where we had used CT examination with a dedicated image analysis tool to assess implant loosening after THR. We compared these results with the results after the standard X-ray examination in these patients with the surgical findings as the true result. The implant loosening tool pilot showed that a provocation-CT-based system has a potential for greatly increased diagnostic precision compared to plain X-ray examination. This together with its much-reduced rate of inconclusive results compared to conventional radiographic examination gave the orthopedic surgeon better decision material in his decision to revise the THR because of implant loosening. It is important to note that the patient population was selected for the provocation-CT (IMA) due to diagnostic difficulties after conventional radiographic assessment, and consequently, they are not representative for the general revision population. In addition, the ability of the loosening tool to accurately diagnose nonloosening could not be looked at with this study, as those patients were generally not operated.

Based on our review of our cases where we had compared provocation-CT (IMA) with plain X-ray examination, the provocation-CT-based approach led to a correct result according to the surgical findings in revision in 14 of the 20 implants compared to 3 of the 20 implants examined with the standard X-ray method. This indicates that a diagnostic tool with high precision could be useful in diagnosis of implant loosening in THR. As to how much displacement is required in order for this method to detect it, experience indicates a threshold around half a millimeter, with significantly smaller displacements down to a third of a millimeter possible to detect but gradually harder to interpret. In our experience, cups and stems are similar in this regard.

In conclusion, we estimated that almost 200 patients in our registry study had mechanical complications after THR, mainly implant loosening, and that a third of these could have been correctly diagnosed and treated if CT examination with a dedicated image analysis tool to assess implant loosening after THR had been available. Our patient review indicated that provocation-CT (IMA) was substantially better than regular plain-X-ray examination at finding the loose implants and met the requirements for such a tool. Future studies could aim at describing the IMA tool in a larger cohort.

## Figures and Tables

**Table 1:** The cause for reimbursement among the 200 scanned patients.

Cause	Number (%)
Infection	68 (34)
Dislocation	12 (6)
Nerve injury	58 (29)
Miscellaneous	33 (16.5)
Mechanical	29 (14.5)

**Table 2:** Individual results from provocation-CT, standard plain X-ray examination, and surgical findings at revision.

Patient	Provocation-CT	Standard plain X-ray examination	Surgical findings at revision
1	Cup not loose	Cup and stem inconclusive	Cup not loose
	Stem loose		Stem loose

2	Cup loose	Cup not loose	Cup loose
	Stem not loose	Stem not loose	Stem not loose

3	Cup inconclusive	Cup and stem inconclusive	Cup not loose
	Stem loose		Stem loose

4	Cup loose	Cup not loose	Cup loose
	Stem not loose	Stem not loose	Stem loose

5	Ring and cup loose	Ring, cup, and stem inconclusive	Ring and cup loose
	Stem not loose		Stem not loose

6	Cup not loose	Cup and stem inconclusive	Cup not loose
	Stem loose		Stem loose

7	Cup loose	Cup loose	Cup and stem loose
	Stem not loose	Stem not loose	

8	Cup and stem inconclusive	Cup and stem inconclusive	Cup and stem not loose

9	Tibia component not loose	Tibia component not loose	Tibia component not loose
	Stem loose	Stem inconclusive	Stem loose

10	Cup loose	Cup and stem inconclusive	Cup loose
	Stem not loose		Stem not loose

**Table 3:** Results after CT provocation and standard X-ray examination according to the surgical findings at revision.

	Provocation-CT Cups (*n* = 9) Stems (*n* = 10) Tibial component (*n* = 1)	Standard X-ray examination Cups (*n* = 9) Stems (*n* = 10) Tibial component (*n* = 1)
Cup- correct result	7	1
Stem- correct result	6	1
Tibial component- correct result	1	1
Cup- inconclusive or incorrect result	2	8
Stem- inconclusive or incorrect result	4	9
Tibial component- inconclusive or incorrect result	0	0

## Data Availability

The data are kept at the LöF and KS by Dr. Gustafson and Olivecrona.

## Supplementary Materials

Supplementary Materials Seeing the degree and nature of loosening in a dynamic provocation-CT facilitates both diagnosis and preparation of revision surgery. The example cup had unclear loosening diagnosis as assessed by plain X-ray and regular static CT.
